# Reduced graphene oxide/zinc oxide composite as an electrochemical sensor for acetylcholine detection

**DOI:** 10.1038/s41598-024-64238-7

**Published:** 2024-06-20

**Authors:** Siraprapa Pitiphattharabun, Krittin Auewattanapun, Thura Lin Htet, Myo Myo Thu, Gasidit Panomsuwan, Ratchatee Techapiesancharoenkij, Jun Ohta, Oratai Jongprateep

**Affiliations:** 1 Department of Materials Engineering, Faculty of Engineering, Kasetsart University, Bangkok, Thailand; 2 Program of Sustainable Energy and Resources Engineering (SERE), Thailand Science Park, TAIST-Tokyo Tech, Pathumthani 12120, Thailand; 3 International Collaborative Education Program for Materials Technology, Education, and Research (ICE-Matter), ASEAN University Network/Southeast Asia Engineering Education Development Network (AUN/SEED-Net), Bangkok, Thailand; 4 Division of Materials Science, Nara Institute of Science and Technology, Nara, Japan

**Keywords:** Reduced graphene oxide, Zinc oxide, Electrocatalytic activity, Acetylcholine, Sensor, Chemistry, Materials science

## Abstract

Acetylcholine (ACh) plays a pivotal role as a neurotransmitter, influencing nerve cell communication and overall nervous system health. Imbalances in ACh levels are linked to neurodegenerative diseases, such as Alzheimer's and Parkinson's. This study focused on developing electrochemical sensors for ACh detection, utilizing graphene oxide (GO) and a composite of reduced graphene oxide and zinc oxide (rGO/ZnO). The synthesis involved modified Hummers' and hydrothermal methods, unveiling the formation of rGO through deoxygenation and the integration of nano-sized ZnO particles onto rGO, as demonstrated by XPS and TEM. EIS analysis also revealed the enhancement of electron transfer efficiency in rGO/ZnO. Cyclic voltammograms of the electrode, comprising the rGO/ZnO composite in ACh solutions, demonstrated prominent oxidation and reduction reactions. Notably, the composite exhibited promise for ACh detection due to its sensitivity, low detection threshold, reusability, and selectivity against interfering compounds, specifically glutamate and gamma-aminobutyric acid. The unique properties of rGO, such as high specific surface area and electron mobility, coupled with ZnO's stability and catalytic efficiency, contributed to the composite's potential in electrochemical sensor applications. This research, emphasizing the synthesis, fabrication, and characterization of the rGO/ZnO composite, established itself as a reliable platform for detecting the acetylcholine neurotransmitter.

## Introduction

Acetylcholine (ACh) is a major excitatory neurotransmitter and neuromodulator in the peripheral and central nervous systems. ACh plays a vital role in transmitting nerve cell signals to other cells in the human body and is involved in various physiological functions, including learning, memory, arousal, attention, and behavioral activities^1–3^. Low concentrations of ACh in the human brain have been associated with neurological disorders such as schizophrenia, Alzheimer's disease, Parkinson's disease, and dementia, while high concentrations can result in reduced heart rate and hypersalivation^4^. Therefore, the detection of ACh is crucial for the diagnosis and treatment of these disorders.

The detection of ACh is typically accomplished using various techniques, such as high-performance liquid chromatography coupled with a fluorometric detection^5^, bioassay combined with a high-performance liquid chromatography^6^, and gas chromatography^7^. However, these techniques are associated with high equipment costs, extensive operator experience, and lengthy sample preparation time^8^. Considering cost-effectiveness, simplicity, selectivity, sensitivity, reusability, and the ability to perform tests near patients (point-of-care testing: POCT), nonenzymatic electrochemical techniques are employed for both in vivo and in vitro ACh detection^9^. To enhance the ACh detection performance of electrochemical sensors, suitable sensing materials are chosen for the working electrodes. Key criteria for selecting appropriate sensing materials include high electron transfer and good sensitivity. Metal oxides have been widely used in sensor applications due to their high catalytic performance and selectivity. Additionally, carbonaceous materials are employed as matrices in metal-oxide carbon composites to further enhance electrocatalysis and achieve synergistic effects on the sensing material's performance^10–12^. Consequently, carbonaceous material/metal oxide composites have been investigated for their high electrocatalytic activity.

Metal oxides have attracted significant attention from materials scientists due to their unique characteristics and properties. One notable metal oxide is nanoparticulate zinc oxide (ZnO), which exhibits exceptional qualities such as nontoxicity, chemical stability, biocompatibility, flexible tunability, and catalytic efficiency^13,14^. ZnO has been extensively utilized in various applications, including antibacterial, biomedical, semiconducting, photocatalytic, supercapacitor, and particularly in electrochemical sensor applications^15–18^. Because of its elevated isoelectric point, which enhances the adsorption mechanism of enzymes and proteins, coupled with its exceptional binding characteristics, ZnO nanoparticles at the nanoscale are regarded as a favorable option for electrochemical sensors^19^. However, the conductivity of ZnO is limited, impeding its electrocatalytic performance^20^.

Graphene oxide (GO) is a two-dimensional sheet of carbon atoms with a hexagonal lattice structure, which contains oxygen functional groups. GO can be prepared through physical methods, including scotch-tape adhesion, micromechanical cleavage, ball milling, and sonication, which are employed for the mechanical exfoliation of graphite^21^. Various chemical methods have also been used for the synthesis of GO. These methods encompass the unzipping of multiwalled carbon nanotubes (MWCNTs) and chemical oxidation approaches such as Brodie's, Staudenmaier's, and Hummers' methods^22,23^. The Hummers' method, known for its high oxidation degree, low toxicity, and short processing time, is a popular choice for the GO synthesis^24^. Because of its distinct characteristics, which encompass a substantial surface area, exceptional electron mobility, and impressive thermal and chemical robustness, graphene oxide (GO) has been widely utilized across diverse sectors like sensor technology, electronics, medical science, and energy storage^25,26^.

The composite of graphene oxide and zinc oxide can be prepared using various methods, including the solvothermal method, microwave irradiation method, and wet chemical method^27–29^. Among these methods, the hydrothermal method is a simple, robust, and efficient technique commonly used for the preparation of the composite^30,31^. Furthermore, the hydrothermal method can be utilized to prepare reduced graphene oxide (rGO). rGO generally exhibits superior electron conductivity and mobility compared to GO due to the reduction of the oxygen content^32,33^. It has been reported that controlling the pH of the precursor during the hydrothermal process enables the reduction of graphene oxide^34–36^. Studies by Bai et al. and Bosch-Navarro et al. have shown that the hydrothermal method under alkaline conditions leads to a decrease in the number of defects and reduced aggregation of rGO sheets^37,38^. During the hydrothermal process of a system consisting of GO and zinc ions, the OH^-^ ions in the solution react with Zn^2+^ ions to form Zn(OH)_2_, resulting in the formation of ZnO nanoparticles on the surface of rGO^39^.

In this regard, the rGO/ZnO composite has demonstrated superior electrocatalytic activity compared to single-phase materials^40,41^. Synergistic effects resulting from the incorporation of rGO and ZnO have been reported. ZnO acts as an activator that reacts with the analyte, and rGO enhances the efficiency of ZnO in electrochemical reactions^42^. Additionally, rGO suppresses the recombination of charge carriers while facilitating high electron transport, thereby leading to enhanced composite efficiency^43^. As a result, the electrocatalytic activity of the rGO/ZnO composite can be significantly improved.

The aim of the present study was to fabricate an rGO/ZnO working electrode for the detection of acetylcholine (ACh). Graphene oxide (GO) was synthesized using a modified version of the Hummers' process, while the rGO/ZnO nanocomposite was prepared through a hydrothermal approach. The potential utilization of the nanocomposite as an electrochemical sensor for ACh detection was evaluated through its electrocatalytic performance, which was measured using cyclic voltammetry and chronoamperometry techniques.

## Experimental

### Synthesis of graphene oxide

GO was synthesized using the modified Hummers' method. A suspension was prepared by combining 1 g (0.08 mol) of graphite powder with an average size of less than 20 µm (Aldrich), 1 g (0.01 mol) of sodium nitrate (NaNO_3_, Kemaus), and 33 mL of sulfuric acid (98% H_2_SO_4_, Merck) to achieve the concentration of 18 mol/L. The suspension was stirred at temperatures below 10 °C until a homogeneous mixture was obtained. Then, 6 g (0.04 mol) of potassium permanganate (KMnO_4_, Kemaus) was slowly added to the mixture. The suspension was then diluted to a concentration of 1.0 mol/L by adding deionized water. With continuous stirring, the temperature of the suspension was raised to 95 °C. Further dilution of the suspension to 0.4 mol/L was achieved by adding deionized water. To stop the reaction and reduce the residual amount of KMnO_4_, 6 mL of 30% H_2_O_2_ was added to the suspension. Centrifugation of the suspension resulted in the formation of a GO precipitate, which was subjected to repeated washing until the pH reached a stable range of 6–7. Finally, the precipitate was dried at 60 °C for 24 h.

### Synthesis of reduced graphene oxide/zinc oxide (rGO/ZnO) composite

The rGO/ZnO composite was synthesized using a hydrothermal technique. In the synthesis process, zinc nitrate hexahydrate (Zn(NO_3_)_2_·6H_2_O, Daejung) and the synthesized GO were dispersed in deionized water at a zinc nitrate-to-GO mass ratio of 2:1. The mixture was sonicated for 30 min to achieve a homogeneous aqueous solution. The pH of the solution was adjusted to a range of 11–12 by adding a 6 M sodium hydroxide solution (NaOH, Pine Chemical), following the method described by Zhou^31^. Subsequently, the solution underwent an autoclave process at 150 °C for 12 h. Afterward, the rGO/ZnO composite powder was obtained by drying the solution at 60 °C for 24 h.

### Preparation of rGO/ZnO composite electrode

**Figure 1:**
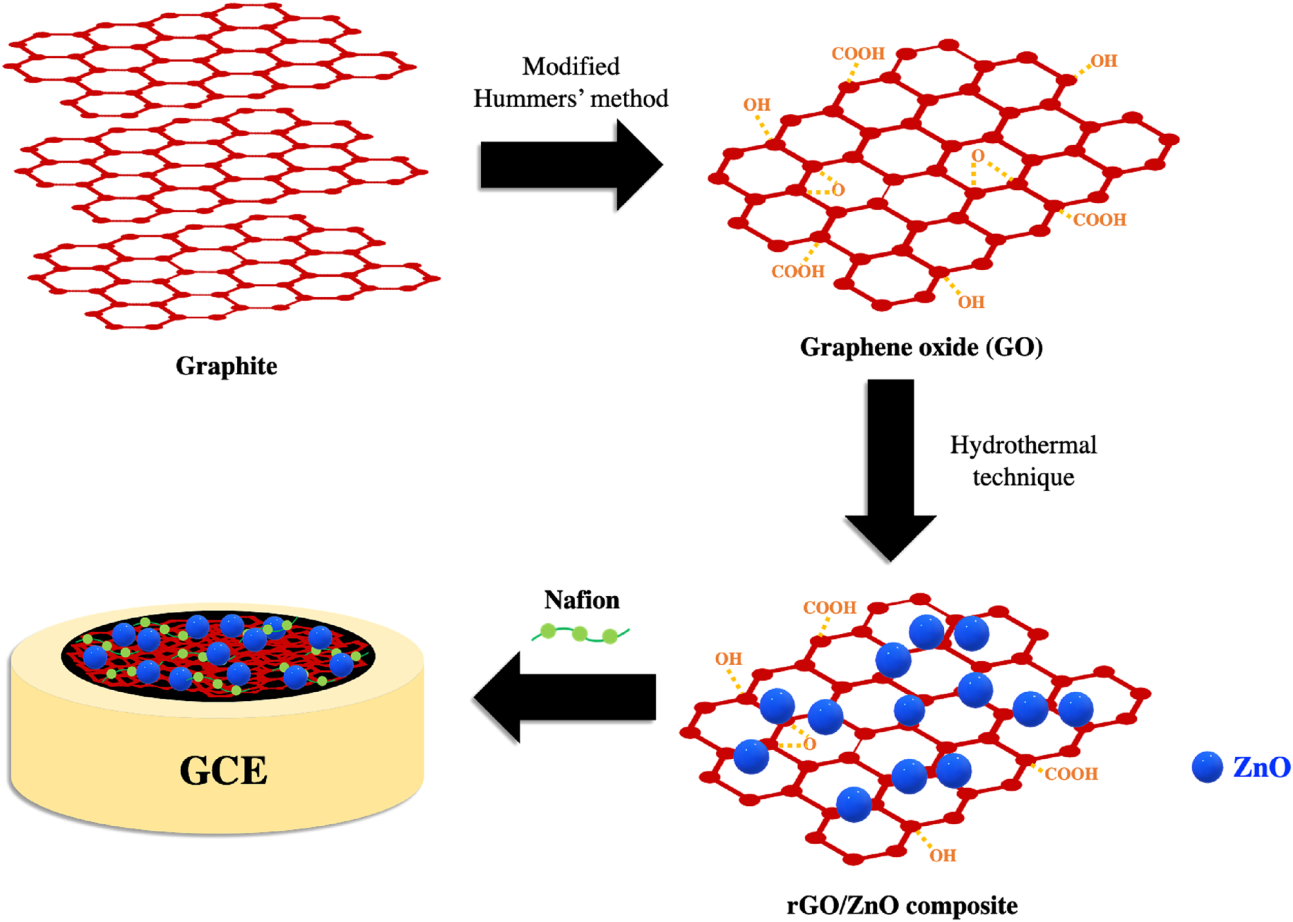
Schematic of the reduced graphene oxide/ZnO composite on a glassy carbon electrode.

Owing to the minimized oxygen functional groups on the surface, rGO exhibits hydrophobic properties, which hinder its dispersion in hydrophilic solutions. To address this issue, Nafion, an organic binder that possesses both hydrophobic and hydrophilic characteristics, is applied to the rGO to enhance its dispersibility in hydrophilic solutions. The rGO/ZnO composite, ethanol, and Nafion (Nafion@perfluorinated resin solution, Aldrich) were mixed in a volumetric ratio of 1:0.95:0.05 (composite:ethanol:Nafion). The mixture underwent 30 min of sonication to attain homogeneity. A glassy carbon electrode (GCE) with a diameter of 3 mm was prepared by sequentially polishing the electrode with diamond and alumina pastes. Subsequently, 5 µL of the rGO/ZnO ethanol and Nafion mixture was dropped onto the electrode and allowed to dry overnight at room temperature, as depicted in Fig. 1.

### Characterization

Phase analysis of the GO and rGO/ZnO composite was conducted by means of X-ray diffraction (XRD; Phillips, X’Pert), over two theta angles ranging from 5° to 80° (step size: 0.02°). In addition to phase identification, the interlayer spacing, stacking height, and number of layers were determined from XRD patterns of the synthesized GO by using the following equations:

1 $$d= \frac{\uplambda }{2\text{sin}\theta }$$

2 $$D = \frac{{K\uplambda }}{{\upbeta \cos \theta }}$$

3 $$n= \frac{\text{D}}{d}+1$$

where, *d* is the interlayer spacing of GO (nm), λ is the wavelength of the X-ray beam (0.154 nm for Cu Kα radiation), θ is the Bragg angle at high intensity, and *D* is the average stack height (nm). Moreover, *K* is a geometry and structure-related factor, which is equal to 0.92 for the determination of the graphene stacking height, β is the full width at half maximum (FWHM) (radians) of the diffraction peak, and *n* is the number of graphene layers.

The morphology of GO and the rGO/ZnO composite was examined using scanning electron microscopy (SEM; FEI, Quanta 450), coupled with energy-dispersive X-ray spectroscopy (EDS; FEI, Quanta 450), and transmission electron microscopy (TEM; HR, JEM-3100).

Raman spectroscopy (NT-MDT, NTEGRA Spectra) was employed to determine the GO structure at room temperature. The spectra were recorded in the wavenumber range of 1000–3200 cm^–1^ using a 532 nm laser.

The specific surface area of GO and the rGO/ZnO composite was determined using a surface area analyzer (Micromeritics, 3Flex) through the Brunauer–Emmett–Teller (BET) technique. Adsorption–desorption isotherms of nitrogen gas at 77 K were obtained after degassing the GO and rGO/ZnO composite at 100 °C for 12 h.

X-ray photoelectron spectroscopy (XPS; ULVAC-PHI, PHI 5000 VersaProbe II) was employed to determine the chemical states, elemental compositions, and functional groups of GO and the rGO/ZnO composite using a monochromatic Al Kα X-ray source (1486.6 eV). The analysis was conducted at pass energy of 117.40 eV and 46.95 eV for wide and narrow scanning, respectively.

To assess the charge transfer capability of the rGO/ZnO and ZnO electrodes, Electrochemical Impedance Spectroscopy (EIS) was employed using a potential impedance analyzer (VSP BioLogic). The setup included rGO/ZnO and ZnO working electrodes, with Ag/AgCl and platinum electrodes serving as the reference and counter electrodes, respectively. EIS measurements were performed with an electrolyte solution consisting of 5 mM potassium ferrocyanide trihydrate K_4_[Fe(CN)_6_] 3H_2_O (Daejung) in 0.1 M potassium chloride KCl (Kemaus). The applied frequency range for the measurements ranged between 200 kHz and 10 mHz, utilizing a 0.2 V applied voltage.

Electrocatalytic activities were evaluated using the cyclic voltammetry technique (Potentiostat machine, Zensor simulator, ECAS 100). Ag/AgCl and Pt electrodes were used as the reference and auxiliary electrodes, respectively. The working electrode for the detection of ACh was a GCE containing the rGO/ZnO composite. Cyclic voltammetry (CV) measurements were conducted at applied voltages ranging from − 1.4 to 1.4 V and scan rates ranging from 25 to 100 mV s^–1^. Prior to the measurement, the working electrode was activated with 0.1 M Na_2_SO_4_. The selectivity of the rGO/ZnO composite electrode in the presence of glutamate (Glu) and gamma-aminobutyric acid (GABA) was determined using the chronoamperometry technique.

### Ethics statement

The research described in the document did not involve experimentation on humans or animals.

## Results and discussion

### Characteristics of GO from modified Hummers’ method

**Figure 2:**
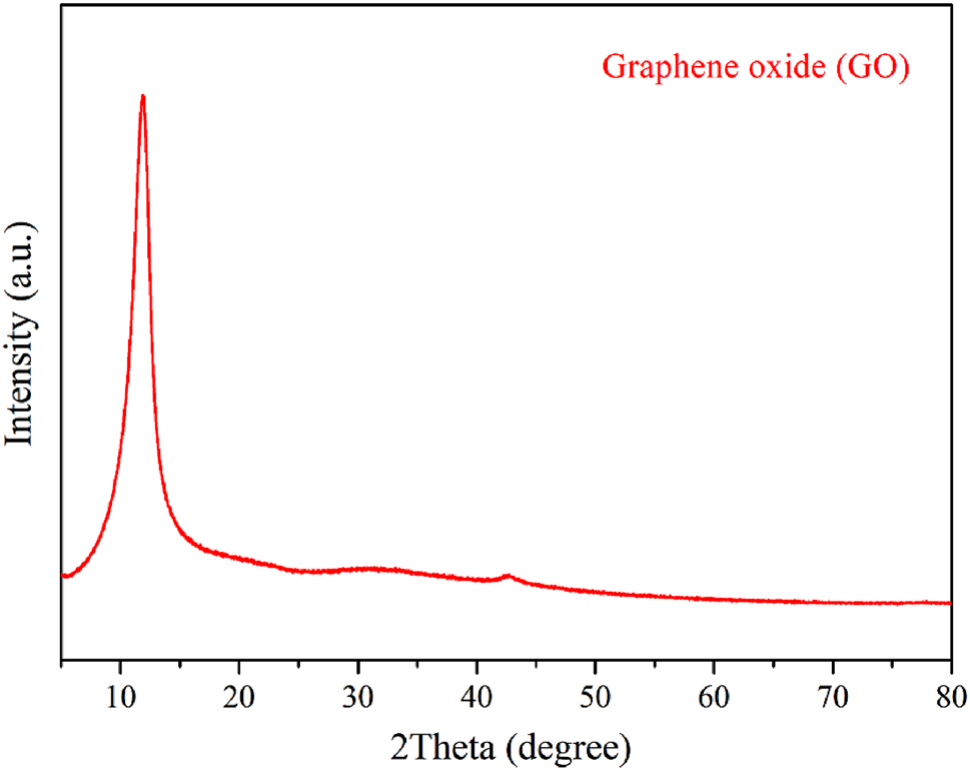
X-ray diffraction pattern of the synthesized GO.

As shown in Fig. 2, the X-ray diffraction (XRD) pattern indicated the successful synthesis of GO using the modified Hummers’ method. The prominent diffraction peak observed at a diffraction angle (2θ) of 11.37° corresponded to GO. Additionally, the interlayer spacing (*d*) of the GO, which was determined to be 0.777 nm using Bragg's equation (Eq. 1), was significantly larger than that of graphite (0.34 nm). This confirmed that the oxidation method successfully expanded the graphite layers. The expansion of the carbon layer is a result of the incorporation of oxygen functional groups on the surface of the GO sheet^44^.

The average crystallite stack height was calculated to be 3.69 nm using Eq. (2), and the number of graphene layers was determined to be close to five using Eq. (3). Previous studies have shown that GO synthesized using the Hummers’ method can have a few-layered structure (3–10 layers) or be multilayered (> 10 layers) with a stack of consecutive carbon sheets^45–47^. The results obtained in this study regarding the number of GO layers and stack height are comparable to those reported in previous studies, as shown in Table 1.

**Table 1:** Interlayer spacing and C/O ratio of GO.

Method	Interlayer spacing (nm)	C/O ratio from EDS	References
Hummers’ method	0.888	1.13	^48^
Improved Hummers’ method	0.703	1.89	^49^
Modified Hummers’ method	0.771	1.79	^50^
Modified Hummers’ method	0.777	1.71	This work

**Figure 3:**
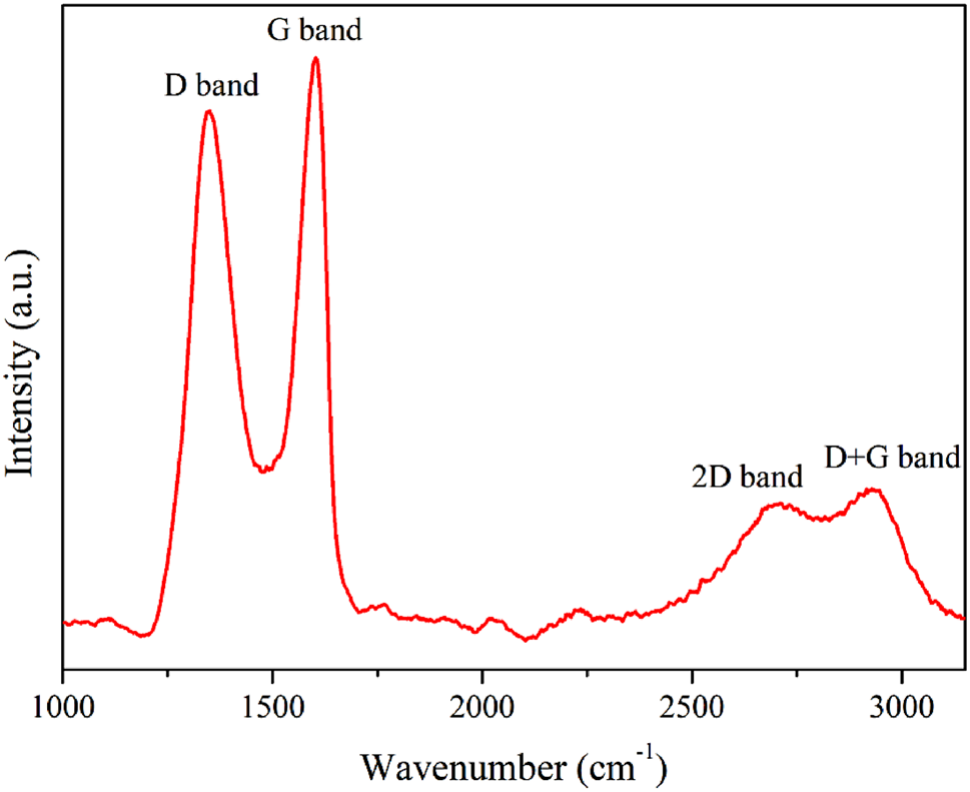
Raman spectrum of GO synthesized using the modified Hummers’ method.

The *sp*^2^ and *sp*^3^ hybridization of carbon atoms in graphite, GO, and rGO was evaluated via Raman spectroscopy. The *sp*^2^ hybridization is indicated by a G band that is related to bonding between carbon and carbon, whereas *sp*^3^ hybridization is indicated by a D band that is associated with oxygen functional groups. Furthermore, the D band and G band peaks occur (in general) at 1350 and 1600 cm^–1^, respectively. In this study, the peaks representing the D band and G band occurred at 1349.59 and 1600.91 cm^–1^, respectively (Fig. 3).

The forms of graphite, GO, and rGO were identified by determining the peak intensity ratio of the D band to the G band (I_D_/I_G_ ratio). I_D_/I_G_ ratios ranging from 0.8 to 1.0 are generally observed for GO^51–55^. In this study, an I_D_/I_G_ value of 0.91 was obtained, confirming the successful production of GO using the modified Hummers’ method. Additionally, broadened peaks of the 2D band and D + G band were observed at 2700.71 and 2939.49 cm^−1^, respectively (Fig. 3). The width of the 2D peak indicates the stacking order of the carbon structure, while the D + G band indicates the presence of defects from oxygen functional groups on the carbon sheet. These results confirm the presence of defects from oxygen functional groups on the GO sheets^56,57^.

**Figure 4:**
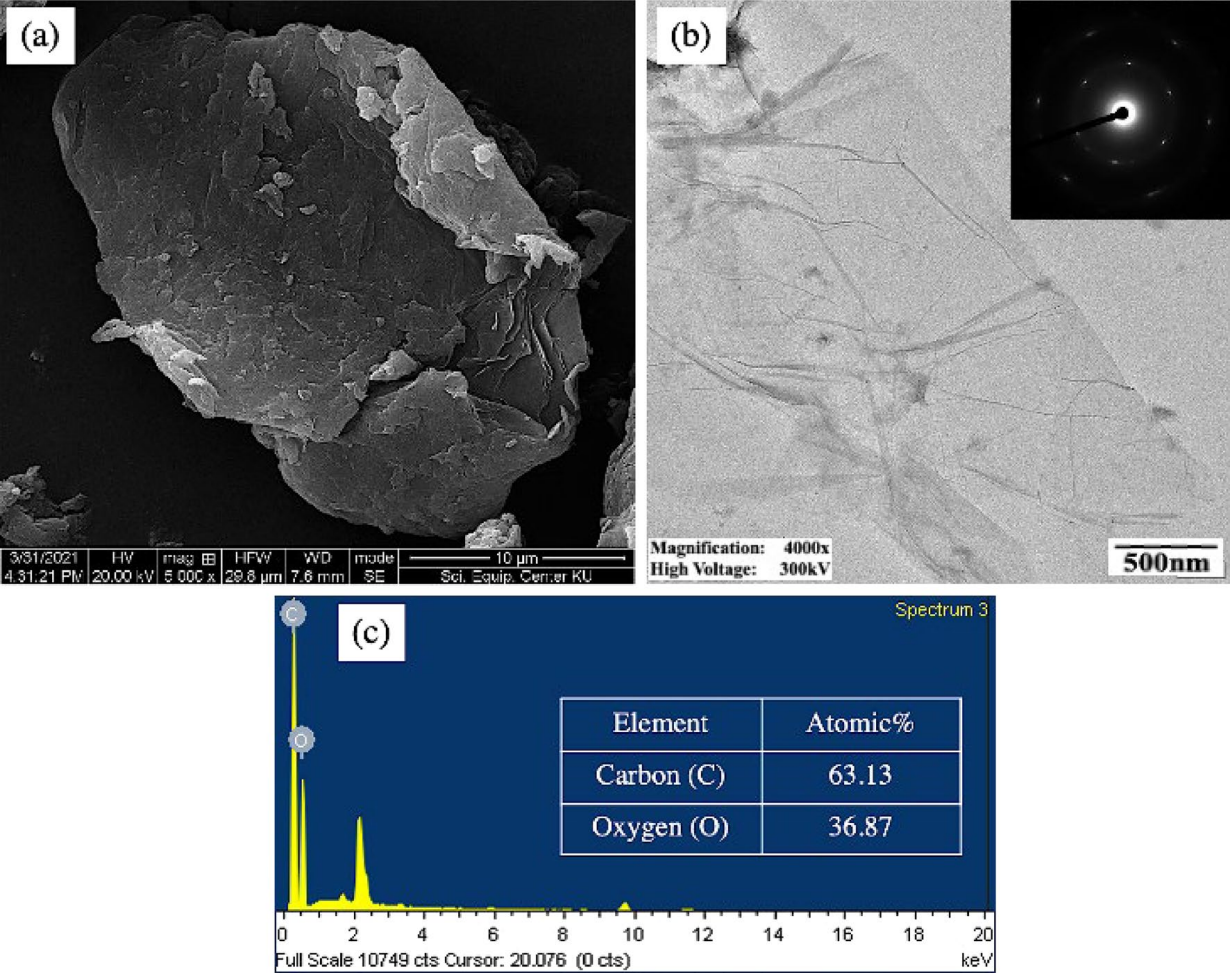
(**a**) SEM micrograph, (**b**) TEM micrograph with electron diffraction pattern, and (**c**) EDS spectrum of the synthesized GO.

As shown in Fig. 4a, an SEM-based morphological study of the synthesized GO revealed flaky and wrinkled stacked sheets. A transmission electron micrograph, as shown in Fig. 4b, revealed a translucent wrinkled sheet, which is consistent with previously reported features^58,59^. According to Aslam et al.^60^, the presence of oxygen functional groups on the surface of GO gave rise to the formation of wrinkled and flawed attributes.

Elemental analysis of the GO was also carried out using EDS, as shown in Fig. 4c. The results revealed prominent peaks of carbon and oxygen, with a content of 63.13 at% C and 36.87 at% O, respectively. The C/O ratio of 1.71 obtained from this study falls within the common range of C/O ratios reported for GO^61–63^. The inset of the TEM micrograph (Fig. 4b) showed an electron diffraction pattern of the synthesized GO. A diffraction pattern, arranged in the form of a hexagonal spot, was distinctly visible. These results corresponded to those of a previously reported few-layered GO^64^. Experimental results obtained from XRD, Raman spectroscopy, SEM, TEM, and EDS confirmed the successful synthesis of GO.

**Figure 5:**
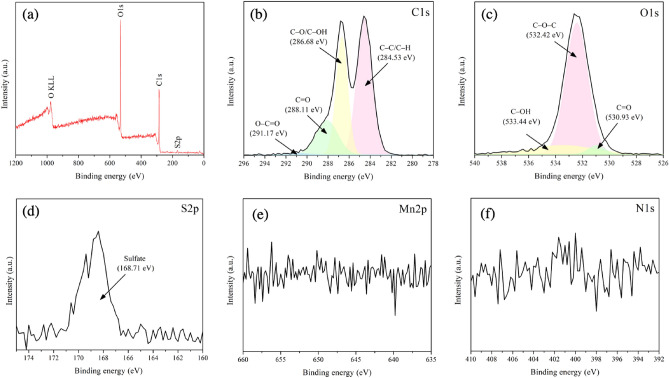
XPS spectra of synthesized GO (**a**) wide scanning and narrow scanning of (**b**) C1*s*, (**c**) O1*s*, (**d**) S2*p*, (**e**) Mn2*p*, and (**f**) N1*s*.

Chemical states and elements of the synthesized GO were also examined via XPS. As shown in Fig. 5a, the wide scanning of GO presented prominent peaks of oxygen (O1*s*) and carbon (C1*s*). Additionally, as shown in Fig. 5d, the wide scanning spectrum also exhibited a low intensity of sulfur (S2*p*) at a binding energy of 168.71 eV, which may be attributed to the remaining sulfur from sulfuric acid (H_2_SO_4_) in the modified Hummers’ method. Despite the sulfur residue, manganese and nitrogen impurities were not detected in the synthesized GO (Fig. 5e and f)^65,66^. The elemental analysis by XPS revealed that the contents of carbon, oxygen, and sulfur were 65.9, 33.6, and 0.4 at%, respectively. The carbon and oxygen content obtained from the XPS analysis were in the comparable range to that obtained from EDS. The sulfate content was in a similar range when compared with other researchers^67–70^.

For high-resolution scanning of C1s, as shown in Fig. 5b, four peaks corresponding to C–C/C–H bonding, C–O/C–OH bonding, C=O bonding, and O–C=O bonding were observed at 284.53, 286.68, 288.11, and 291.17 eV, respectively. The results revealed bonding between carbon and oxygen, which is consistent with the literature that reports structures of graphene oxide fields^71–73^. Furthermore, as shown in Fig. 5c, the high-resolution scanning of O1s reveals peaks at 530.93, 532.42, and 533.44 eV, which correspond to the carbonyl group (C=O), epoxide group (C–O–C), and hydroxyl group (C–OH), respectively.

### Characteristics of composite from hydrothermal method

**Figure 6:**
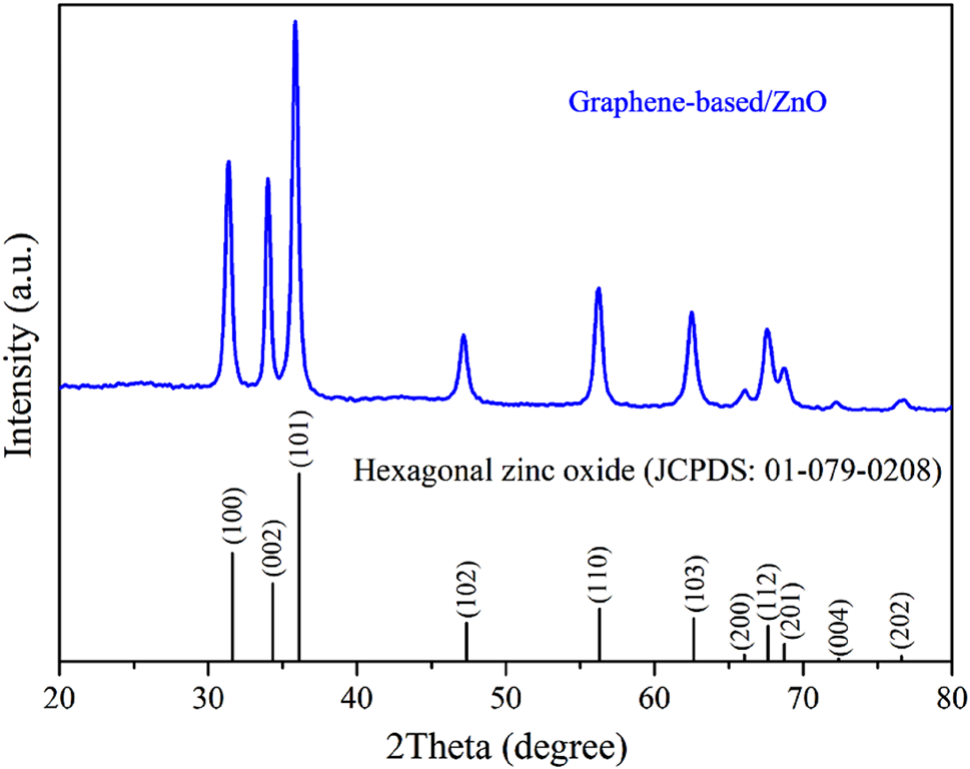
X-ray diffraction pattern of graphene-based/ZnO composite.

In the fabrication of the sensing material for the working electrode, a composite of graphene-based/ZnO was prepared through a hydrothermal treatment of zinc nitrate and GO. The XRD patterns of the composite are shown in Fig. 6. The diffraction pattern shows a prominent peak of hexagonal ZnO (JCPDS 01-079-0208). No diffraction peaks corresponding to a secondary phase were detected, indicating the formation of a single-phase ZnO. However, due to the high intensity of the ZnO peaks and the low aggregation of graphene-based sheets in the composite form, the diffraction pattern of graphene-based material was not clearly observed^74,75^.

**Figure 7:**
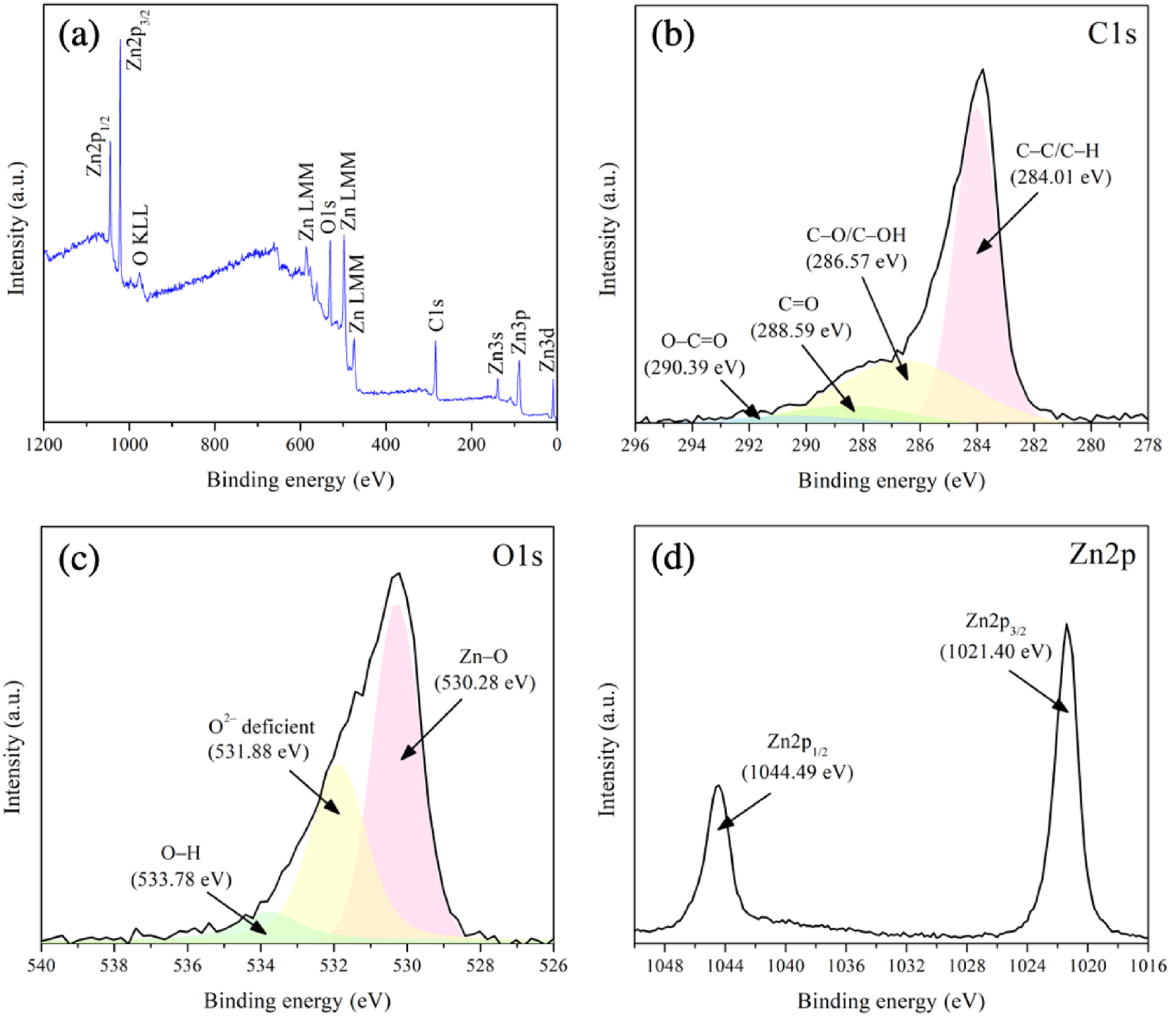
XPS spectra of rGO/ZnO composite (**a**) wide scanning and narrow scanning of (**b**) C1*s*, (**c**) O1*s*, and (**d**) Zn2*p*.

With the indistinct X-ray diffraction peak of graphene-based material, the composite was further investigated by XPS. As shown in Fig. 7a, the wide scanning exhibits prominent peaks of carbon (C), oxygen (O), and zinc (Zn). No evidence of impurity elements was observed. The contents of carbon, oxygen, and zinc from XPS analysis were 50.9, 33.2, and 15.9 at%, respectively.

For the high-resolution spectra of C1s, as shown in Fig. 7b, the peaks representing C–C/C–H bonding, C–O/C–OH bonding, C=O bonding, and O–C=O bonding were detected at 284.01, 286.57, 288.59, and 290.39 eV, respectively. The C1s spectra demonstrate the formation of GO. Nevertheless, the intensity of peaks corresponding to C–O/C–OH bonding, C=O bonding, and O–C=O bonding was clearly minimized. This indicates the deoxygenation of oxygen functional groups, resulting in the formation of rGO^76,77^.

Despite the demonstration in the preceding section that the modified Hummers’ method produced carbon-based materials exhibiting graphene oxide (GO) characteristics, the XPS findings presented in this section indicated that the subsequent hydrothermal process had the capability to transform GO into rGO. This finding aligns with earlier research suggesting the involvement of a hydrothermal process in reducing graphene oxide and generating rGO^78,79^.

The mechanisms associated with the deoxygenation of GO in the presence of zinc nitrate under hydrothermal conditions can be explained as follows: as graphene oxide (GO), containing carbonyl groups, epoxide groups, and hydroxyl functional groups, is dispersed in an aqueous medium under elevated temperature and pressure conditions, the oxygen-containing functional groups of GO interact with Zn^2+^ ions. These functional groups serve as nucleation sites for ZnO nanoparticles. Continuous growth of ZnO crystals takes place on the GO surface, resulting in the formation of ZnO nanoparticles on the GO sheets. Concurrently, due to the interaction between oxygen from GO and Zn^2+^, the oxygen content within the GO sheets undergoes reduction, leading to the formation of reduced graphene oxide.

The XPS analysis of the composite also included the examination of Zn and O spectra. Figure 7d displays the Zn2*p* spectra of ZnO, revealing peaks at 1021.40 and 1044.49 eV, corresponding to Zn2*p*_3/2_ and Zn2*p*_1/2_, respectively. The gap between the Zn2*p*_3/2_ and Zn2*p*_1/2_ peaks measures around 23 eV, a value consistent with findings from other research groups^80–82^.

Figure 7c shows the O1*s* spectra of the composite, indicating the presence of lattice oxygen (Zn–O) and oxygen vacancy peaks at 530.28 and 531.88 eV, respectively. These species are commonly observed in hexagonal ZnO structures^83,84^. Additionally, an O1*s* peak at 533.78 eV was observed, corresponding to hydroxyl groups (–OH) adsorbed on the sample's surface. According to Yu et al.^20^ the adsorption of hydroxyl groups can enhance electrocatalytic performance.

**Figure 8:**
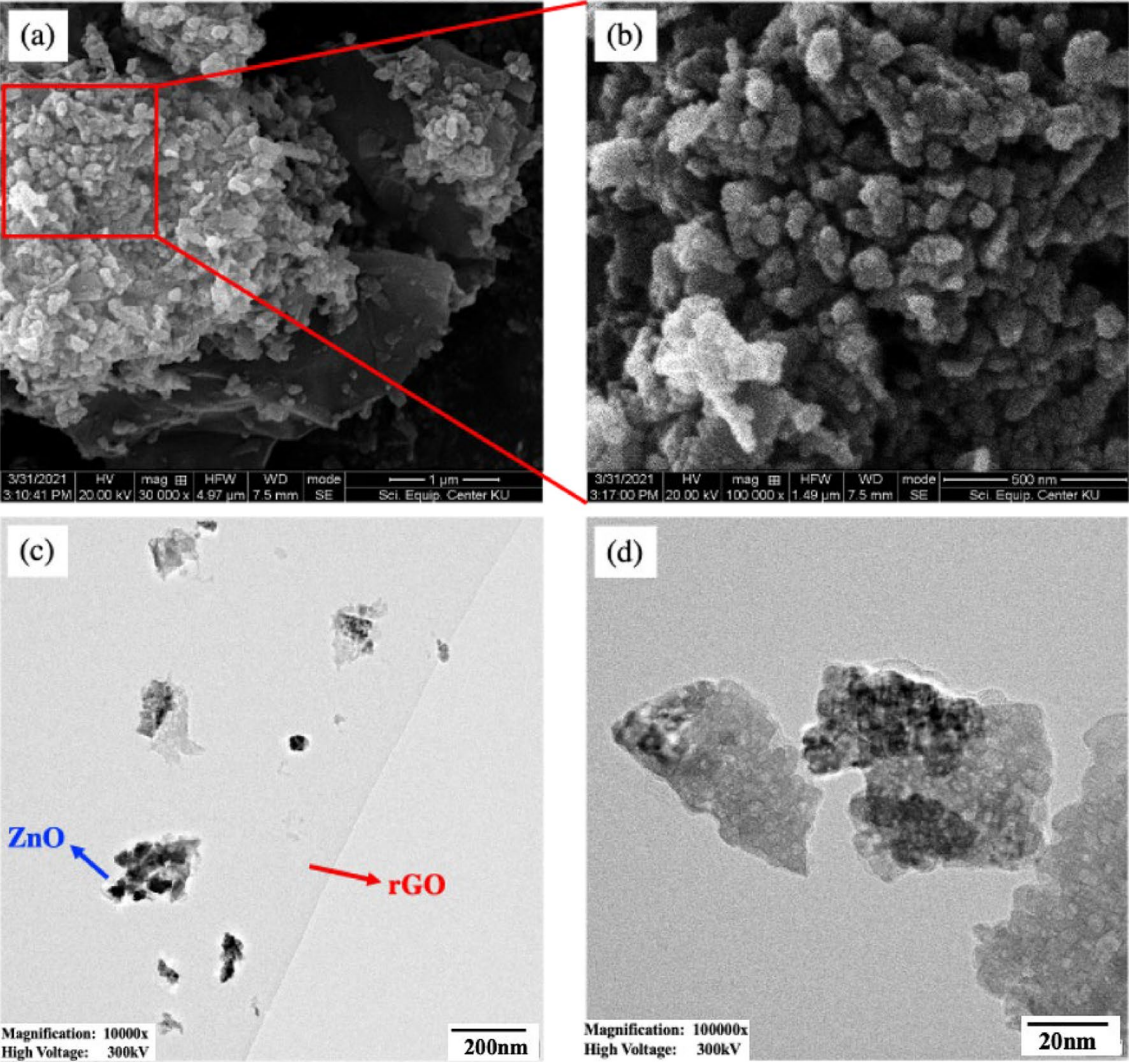
(**a**–**b**) SEM micrographs and (**c**–**d**) TEM micrographs of the (**a**, **c**) rGO/ZnO composite and (**b**, **d**) ZnO particles on surface of rGO.

SEM micrographs, as shown in Fig. 8a and b, reveal the entrapment of ZnO particles on the surface of the rGO. TEM micrographs, as shown in Fig. 8c and d, indicate that the thin-layered rGO sheet is decorated by agglomerated ZnO, which occurs in the form of irregular-shaped particles. The evaluation of SEM and TEM micrographs yielded ZnO average particle sizes of 25.62 ± 2.97 nm and 14.93 ± 3.70 nm, respectively. Analysis based on Scherrer's equation yielded an average ZnO crystallite size of 20.64 nm.

Specific surface area (SSA) measurements were performed using the BET technique to determine the area of active sites for catalytic activities. High SSA is generally associated with an enhanced catalytic performance^85^. The SSA values of GO and the rGO/ZnO composite were found to be 132.79 and 66.02 m^2^/g, respectively. The lower SSA of the composite, compared to that of GO, can be attributed to the agglomeration of ZnO particles and their coverage of the surface of the graphene-based material. According to Babitha et al., the pores of rGO were overlaid by ZnO particles, resulting in a reduction in the SSA of the graphene-based/ZnO composite^86^.

### Electrochemical properties and electrocatalytic activity of rGO/ZnO composite in ACh

**Figure 9:**
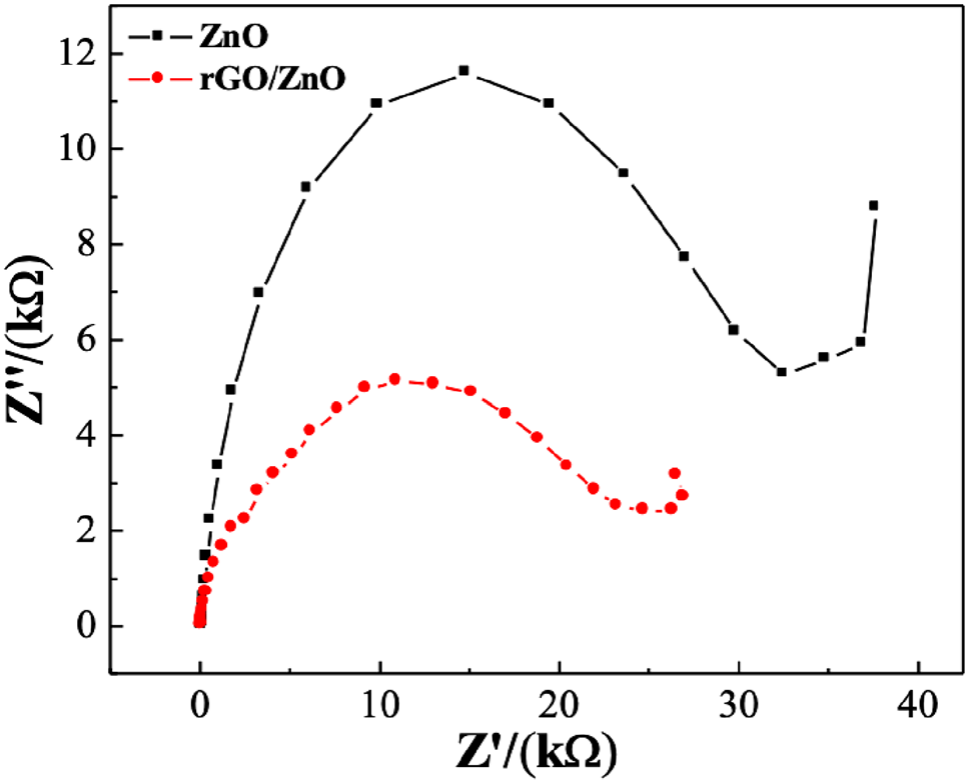
Nyquist plots rGO/ZnO composite electrode and ZnO electrode in a solution of 5 mM of [Fe (CN)_6_]^3−/4−^ in 0.1 M KCl with the frequency ranging from 10 mHz to 100 kHz with applied voltage of 0.2 V.

Electrochemical impedance spectroscopy (EIS) investigations provide valuable insights into the kinetics of electron transfer processes occurring at the electrode–electrolyte interface on a working electrode^87^. In this study, EIS measurements were conducted on glassy carbon electrodes (GCE) decorated with rGO/ZnO and ZnO in a solution containing 5 mM standard potassium ferricyanide in 0.1 M KCl. The assessments, covering a frequency range from 200 kHz to 10 mHz at an applied voltage of 0.2 V, revealed a Nyquist plot illustrating the charge transfer resistance (R_ct_) values for rGO/ZnO and ZnO as 25 kΩ and 37 kΩ, respectively, as depicted in Fig. 9. The findings revealed an increased electrical conductivity in the rGO/ZnO composite electrode. These results from EIS suggest that incorporating rGO in the sensing materials can enhance electron transfer efficiency between the electrode and electrolyte, thereby improving electrocatalytic activity.

To assess the electrocatalytic activities of the rGO/ZnO composite electrode, cyclic voltammetry was employed. The electrode underwent electrochemical activation before conducting cyclic voltammetry measurements of the target analyte. This step was essential for cleaning the electrode surface and removing contaminants that could potentially interfere with the electrochemical reactions under investigation, ensuring accurate measurements, and enhancing the electrode’s sensitivity and selectivity toward the target analyte. Moreover, this activation step promoted reproducibility by standardizing the electrode surface, reducing variability between measurements, and guaranteeing consistent results. In this study, the activation process involved applying potential cycling from − 1.5 to 1.5 V for 1 min to ensure the effective removal of contaminants and to prepare the electrode for subsequent electrochemical measurements, thereby enhancing reliability and reproducibility.

**Figure 10:**
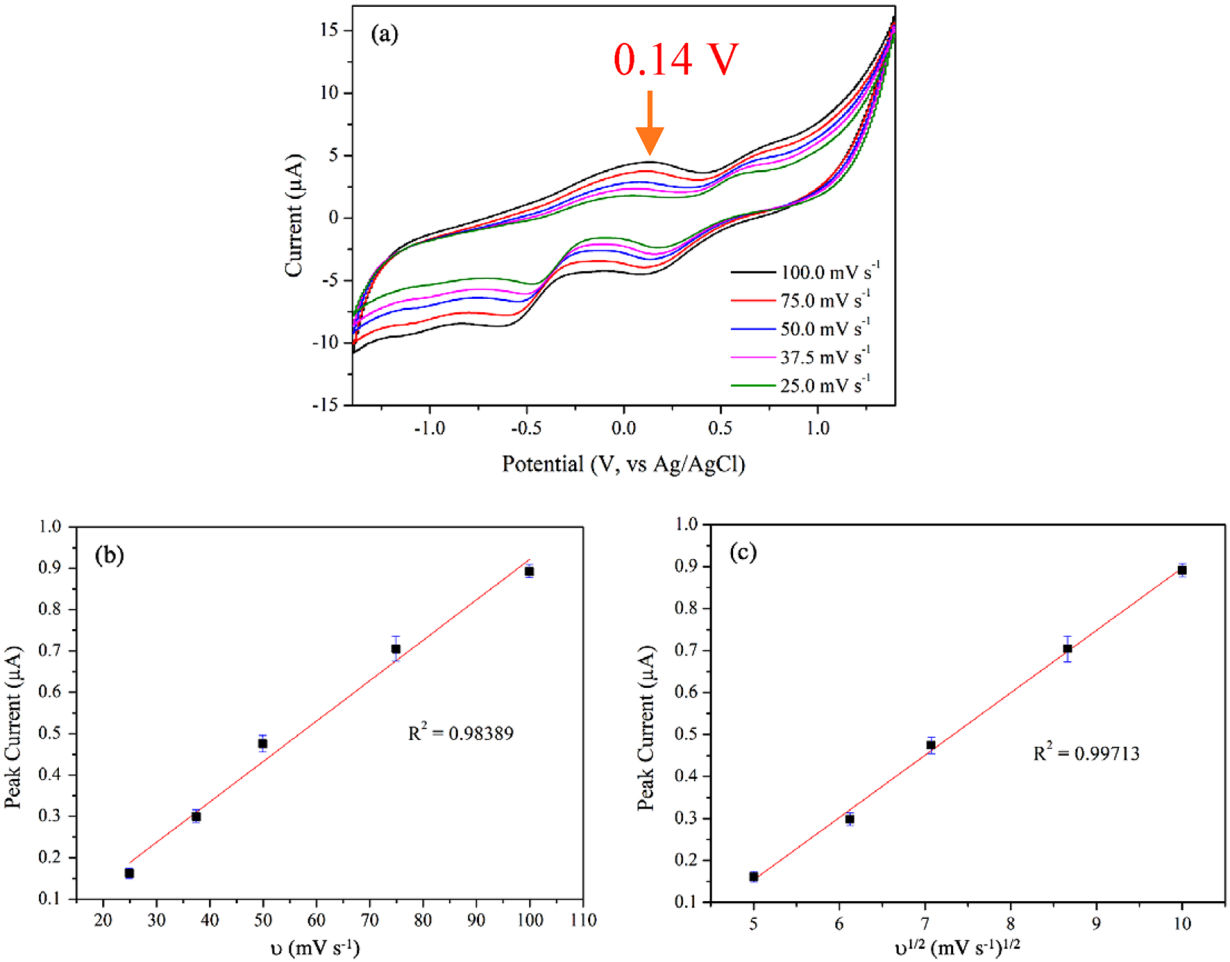
(**a**) Cyclic voltammogram of the rGO/ZnO composite electrode utilized for detecting 500 µM ACh across scan rates ranging from 25.0 to 100.0 mV s^−1^, (**b**) Relationship between the peak current and the scan rate of the oxidation reaction at an applied voltage of 0.14 V, and (**c**) Relationship between the square root of the scan rate and the peak current at 0.14 V.

The measurements, conducted in 500 µM ACh at different scan rates of 25.0–100.0 mV s^−1^, revealed reactions at voltage 0.14 V, which corresponded to the potential at which acetylcholine undergoes oxidation. The reduction reactions occurred at applied voltage of − 0.65 V (see Fig. 10a). The redox mechanisms of ACh on rGO/ZnO could be associated with the interfacial interactions, occurring between the electrode materials and the analytes^88^. According to Hussain et al., the mechanisms of electrocatalytic activity of ZnO·CuO nano-leaves in the presence of acetylcholine involve (1) dissociation of acetylcholine solution into acetic acid and choline, (2) conversion of choline into betaine and hydrogen peroxide (H_2_O_2_), and (3) conversion of hydrogen peroxide (H_2_O_2_) into oxygen and protons^89^. These mechanisms could be described by the following equations.

4 $${\text{Acetylcholine}} + {\text{H}}_{{2}} {\text{O}} \to {\text{Acetic}}\;{\text{ acid }} + {\text{Choline}}$$

5 $${\text{Choline}} + {\text{H}}_{{2}} {\text{O}} + {\text{2O}}_{{2}} \to {\text{Betaine}} + {\text{2H}}_{{2}} {\text{O}}_{{2}}$$

6 $${\text{2H}}_{{2}} {\text{O}}_{{2}} \to {\text{2O}}_{{2}} + {\text{4H}}^{ + } + {\text{4e}}^{ - }$$

In addition to mechanisms related to redox reactions, it is important to address those associated with interface reactions (adsorption) and bulk reactions (diffusion). To investigate these mechanisms, examining the relationships between the peak current and both the scan rate and the square root of the scan rate can provide insights into the nature of the electrochemical processes. A linear relationship between the peak current (i_p_) and the scan rate (ν) indicates a surface-controlled electrochemical process, primarily involving the deposition of analyte on the electrode surface. In this case, the reaction kinetics are mainly determined by the active sites on the electrode, suggesting strong adsorption of reactants. Conversely, a linear relationship between the peak current and the square root of the scan rate indicates a diffusion-controlled process, where the reaction kinetics are governed by the rate at which electroactive species diffuse to the electrode surface.

**Figure 11:**
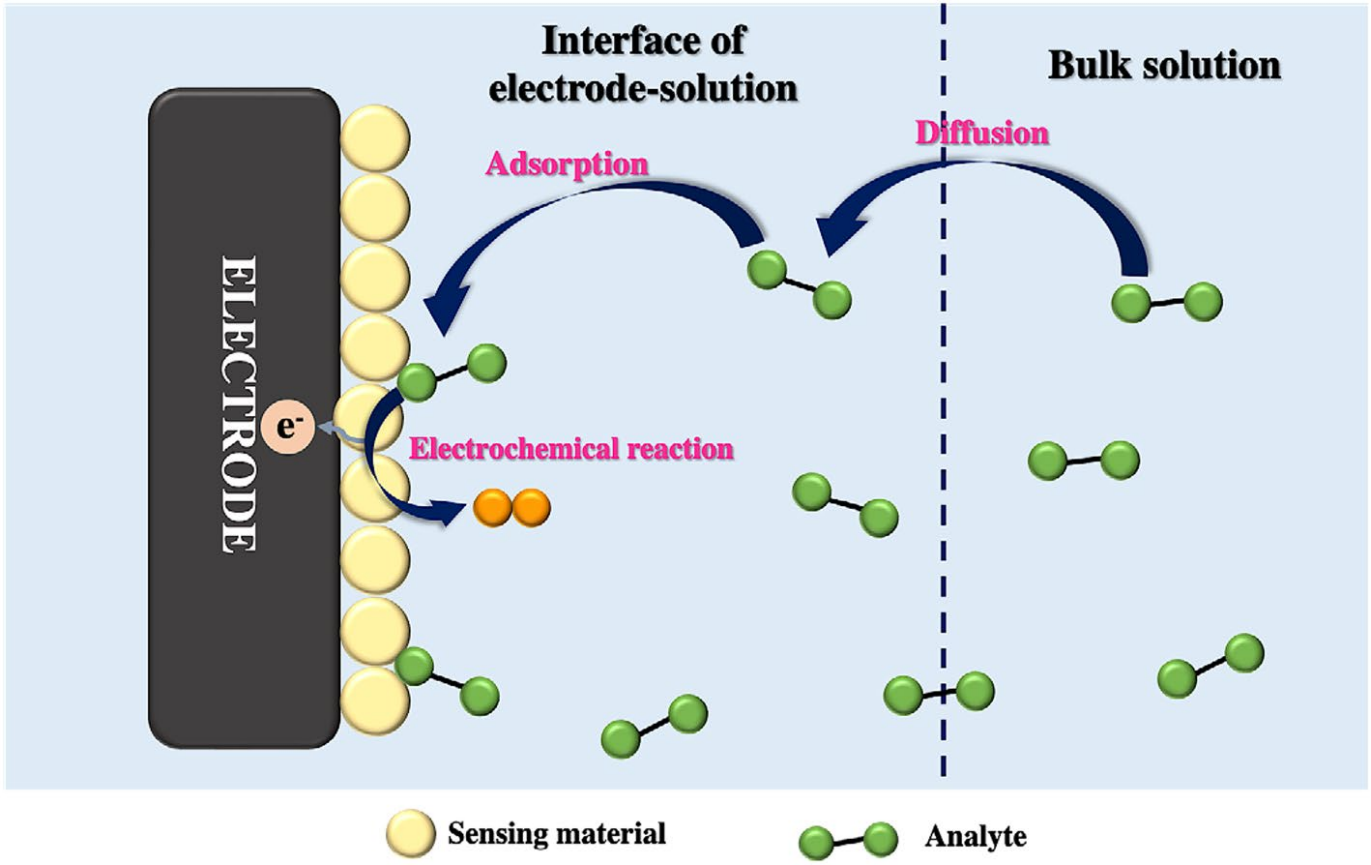
Schematic representation of the diffusion, adsorption, and electrochemical reaction processes between the sensing material and analyte.

In this study, as illustrated in Fig. 10b and 10c, a strong linear dependence was observed for both the scan rate (R^2^ = 0.984) and the square root of the scan rate (R^2^ = 0.997). These results suggested that the electrocatalytic activities of the rGO/ZnO composite electrode in ACh were controlled by both diffusion and adsorption reactions. A schematic representation of the diffusion, adsorption, and electrochemical reaction processes between the sensing material and the analyte is shown in Fig. 11^91,92^.

It should be noted that the CV curve at varying scan rates, illustrated in Fig. 10a, also demonstrated that changes in scan rate were accompanied by slight shifts in both anodic and cathodic peak currents. The observed shift in oxidation and reduction peaks as the scan rate increases—where higher rates induce a positive shift in oxidation potential and a negative shift in reduction potential—could be ascribed to diverse dynamics within the electrochemical system. Firstly, elevated scan rates diminish the timeframe for electron transfer, impacting the electrochemical reaction's capacity to match swift potential variations, consequently leading to shifts in peak potentials. Secondly, the scan rate impacts the thickness of the diffusion layer, with higher rates leading to thinner layers, altering the concentration gradient near the electrode, and influencing peak potentials. Additionally, at an equal interval time, a higher scan rate yields greater electrochemical reactions compared to a lower scan rate. This increase in electrochemical reaction necessitates a higher applied voltage to accommodate the reaction^90^. These factors collectively explained the observed shifts in oxidation and reduction peaks as scan rates increased.

**Figure 12:**
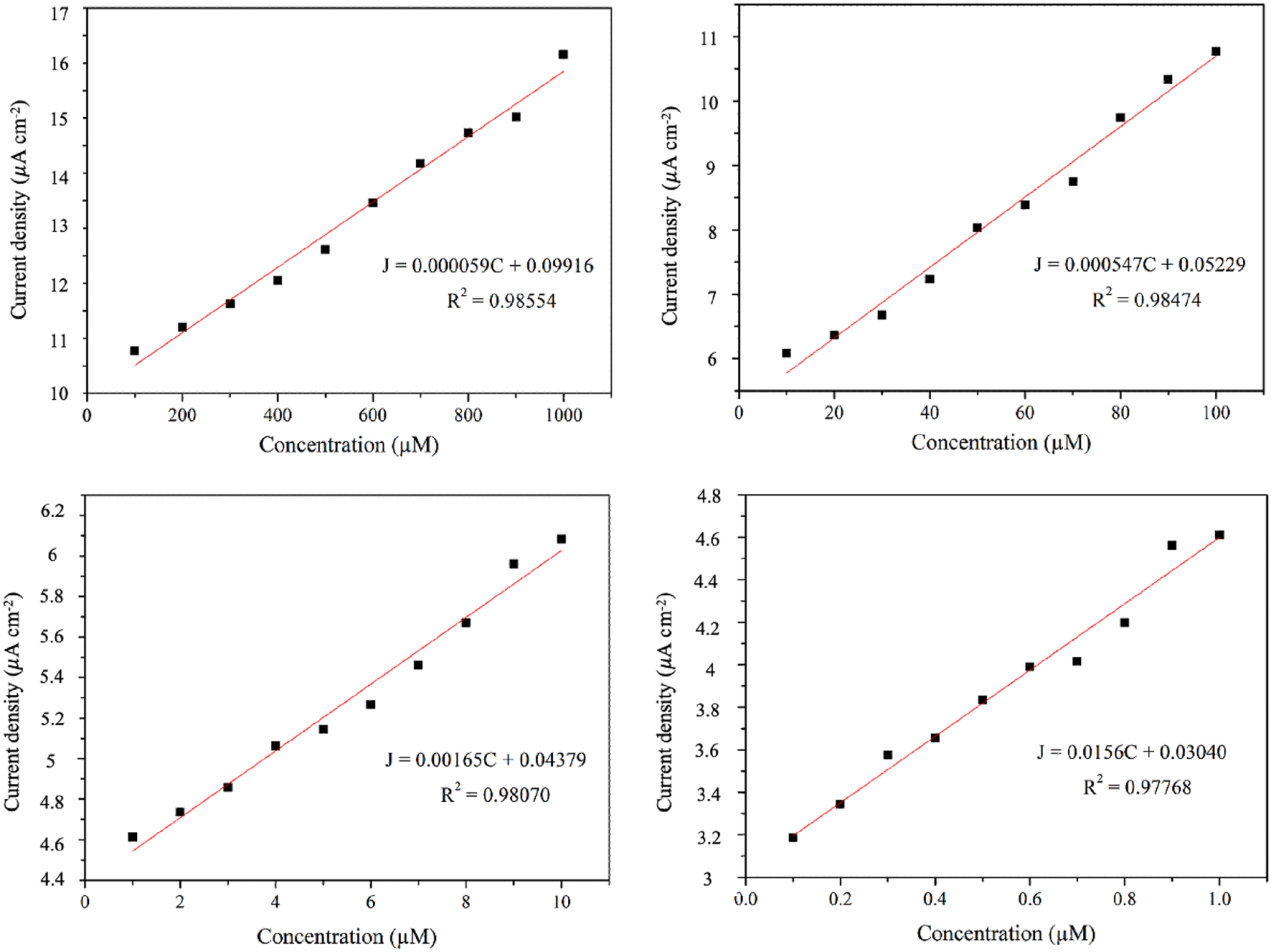
Analytical curve corresponding to the oxidation reaction of rGO/ZnO composite electrode in 0.1–1000 µM acetylcholine at a scan rate of 100.0 mV s^−1^ (The current density was determined at an applied voltage of 0.14 V).

The sensitivity of the rGO/ZnO composite electrode in the detection of ACh was determined from an analytical curve (current density vs. concentration). As shown in Fig. 12 and Table 2, the current density exhibited a strong linear dependence on the concentration for levels ranging from 0.1 to 1000 µM (corresponding sensitivity values ranged from 0.0156 to 0.000059 µA µM^−1^ mm^−2^).

**Table 2:** Sensitivity of rGO/ZnO composite electrode in 0.1–1000 µM acetylcholine.

Sample	Concentration (µM)	Sensitivity (µA µM^−1^ mm^−2^)	*R*^2^
rGO/ZnO composite	100–1000	5.90 × 10^−5^	0.986
	10–100	5.47 × 10^−4^	0.985
	1–10	1.65 × 10^−3^	0.981
	0.1–1	1.56 × 10^−2^	0.978

The limit of detection (LOD), an indicator of sensor performance, reveals the lowest concentration that can be detected at 95% confidence level^93^. The LOD value was calculated as follows:

7 $$LOD= \frac{3.3\sigma }{S}$$

where *σ* is the standard deviation of the blank measurements (n = 10) and *S* is the slope of the analytical curve.

A LOD value of 0.063 µM was obtained for the rGO/ZnO composite electrode detection of ACh. The value lies in the range reported by other researchers, as shown in Table 3.

**Table 3:** Comparison of sensors used for acetylcholine detection.

Sensors	Range of detection (µM)	Sensitivity (µA µM^−1^ mm^−2^)	LOD (µM)	References
Lichen-like NiO/CPE^a^	250–5880	3.92 × 10^−3^	26.7	^94^
Flower-like NiAl-LDHs/CD/GCE^b^	5–6800	1.33 × 10^−3^	1.7	^95^
Ni nanowire array electrode	–	1.40 × 10^−2^	0.84	^96^
rGO/ZnO composite/GCE	0.1–1000	5.90 × 10^−5^–1.56 × 10^−2^	0.063	This work

^a^Lichen-like NiO/CPE = Lichen-like nickel oxide nanostructure modified carbon paste electrode.

^b^Flower-like NiAl-LDHs/CD/GCE = Flower-like NiAl layered double hydroxides decorated with carbon dots modified glassy carbon electrode.

Glutamate, a key neurotransmitter that stimulates activity in the central nervous system, is often present in biological samples and could disrupt the detection of acetylcholine (ACh). Additionally, gamma-aminobutyric acid (GABA), the primary inhibitory neurotransmitter in the central nervous system, along with glutamate (Glu), poses potential obstacles for precise ACh measurements due to their common occurrence in neural tissues. Consequently, the decision to test with glutamate and GABA was made to assess selectivity performance.

**Figure 13:**
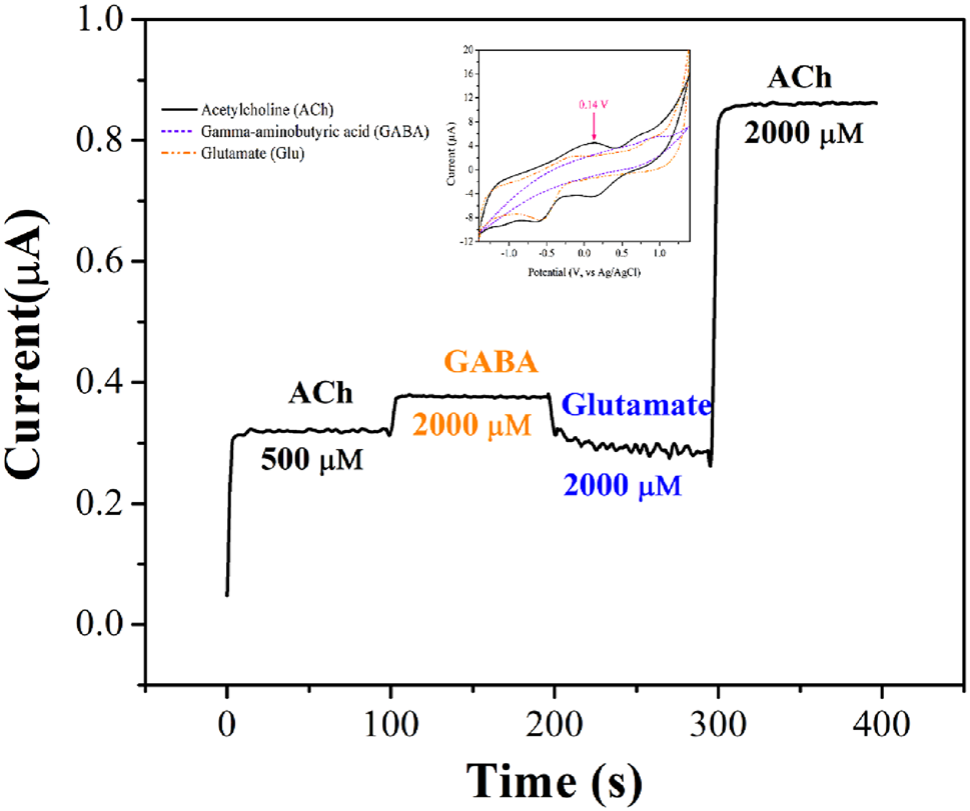
Selectivity of rGO/ZnO composite electrode, measured in 500 µM acetylcholine (ACh), 2000 µM glutamate (Glu), 2000 µM gamma-aminobutyric acid (GABA), and 2000 µM of (ACh) at an applied voltage of 0.14 V. The inset shows cyclic voltammograms of rGO/ZnO electrodes in the presence of ACh, Glu, and GABA.

The chronoamperometry experiments were conducted at an applied voltage of 0.14 V. As depicted in Fig. 13, when 500 µM ACh was introduced into DI water, the rGO/ZnO composite electrode exhibited noticeable peaks in its response. However, when the interference species GABA and Glu were successively added at concentrations ten times higher, specifically 2000 µM, no distinct peak was discerned. Subsequently, after measuring the interference species, the addition of ACh at concentrations of 2000 µM elicited a clear response^97,98^. In summary, the chronoamperometry findings indicated that the current response remained largely unchanged following the introduction of GABA and Glu, while prominent responses were observed in ACh measurement at both concentrations of 500 µM and 2000 µM. This suggested that the sensor possessed high selectivity in detecting ACh, even when commonly interfering substances were present at concentrations ten times higher than that of ACh.

**Figure 14:**
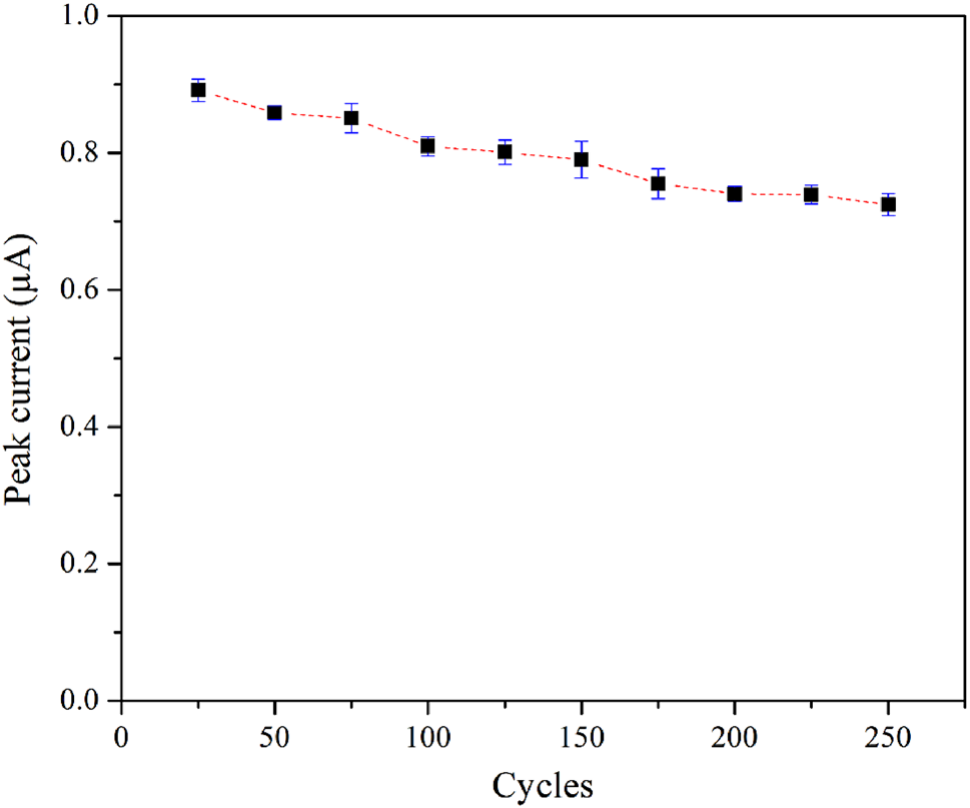
Reusability of rGO/ZnO composite electrode in 500 µM ACh measured at a scan rate of 100.0 mV s^−1^.

As shown in Fig. 14, the reusability of the rGO/ZnO composite electrode for the detection of ACh was examined. The results revealed that the current response remained > 91% after the electrode was used for 100 cycles of measurement. After 250 measurement cycles, the remaining current response was 80%. These results indicate fair reusability of the electrode in detecting ACh.

## Conclusion

A few-layer GO was successfully synthesized using the modified Hummers’ method, while a rGO/ZnO nanocomposite was effectively prepared via a hydrothermal method. The nanocomposite was used as the sensing material in the working electrode, and its electrocatalytic performance was evaluated. Cyclic voltammetry (CV) was performed in the presence of ACh, resulting in prominent oxidation and reduction reactions observed at applied voltages close to 0.14 V, 0.72 V, 0.09 V, and -0.65 V. The analytical curve of the rGO/ZnO electrode exhibited good linearity for ACh concentrations ranging from 0.1 to 1000 µM. With acceptable sensitivity (1.56 × 10^−2^–5.90 × 10^−5^ µA µM^−1^ mm^−2^), decent ACh selectivity in the presence of Glu and GABA, and satisfactory reusability, the rGO/ZnO nanocomposite showed great potential for ACh detection.

## Data Availability

The data that support the findings of this study are available upon reasonable request to Dr.Oratai Jongprateep (fengotj@ku.ac.th).
